# Assessing the future global distribution of land ecosystems as determined by climate change and cropland incursion

**DOI:** 10.1007/s10584-023-03584-3

**Published:** 2023-07-28

**Authors:** Richard D. Robertson, Alessandro De Pinto, Nicola Cenacchi

**Affiliations:** 1 International Food Policy Research Institute, Washington, DC USA; 2 University of Greenwich, Chatham, Kent UK

**Keywords:** Land use, Land cover, Climate change, Cropland, Agriculture

## Abstract

The geographic distribution of natural ecosystems is affected by both climate and cropland. Discussions of future land use/land cover usually focus on how cropland expands and displaces natural vegetation especially as climate change impacts become stronger. Less commonly considered is the direct influence of climate change on natural ecosystems simultaneously with cropland incursion. We combine a natural vegetation model responsive to climate with a cropland allocation algorithm to assess the relative importance of climate change compared to cropland incursion. Globally, the model indicates that climate change drives larger gains and losses than cropland incursion. For example, in the Amazonian rainforests, more than one sixth of the forest area could be lost due to climate change with cropland playing virtually no role. Our findings suggest that policies to protect specific ecosystems may be undercut by climate change and that localized analyses that fully account for the impacts of a changing climate on natural vegetation and agriculture are necessary to formulate policies that preserve natural ecosystems over the long term.

**Supplementary Information:**

The online version contains supplementary material available at 10.1007/s10584-023-03584-3.

## Introduction

Climate change and an evolving human demand for food products will drastically affect how much land can be occupied by natural ecosystems in the future. As population and incomes grow, more agricultural area might be required to meet human demands at the expense of natural ecosystems (FAO 2018). Such expansion of agriculture will be exacerbated as agricultural productivity is threatened by increasing temperatures, changing precipitation patterns, and a higher incidence of extreme weather events (Nelson et al. 2014).

Assessing how these factors influence the future encroachment of agriculture into sensitive natural biomes is the driving motivation behind many global models that investigate the future trajectories of land use and land cover (LULC). Recently, interest in LULC projections has increased because of the threat of the release of the carbon stock previously locked in soils and in natural environments such as tropical forests and tundra. To investigate issues at the nexus of climate change, bio-geophysical, and socio-economic phenomena, several studies bring together Earth system and integrated assessment models which include LULC modeling components (Bond-Lamberty et al. 2014; Hasegawa et al. 2017). The resulting projections for future changes in LULC vary significantly across studies. For example, some estimates indicate that by 2050, cropland might grow by 125–400 Mha, forest losses could range from 87 to 250 Mha, and pastureland could decrease by 150–289 Mha (Hasegawa et al. 2017; Dietrich et al. 2019; Kreidenweis et al. 2018; Schmitz et al. 2014). Estimates for changes in other natural land (i.e., all other land types besides cropland, pasture, bioenergy cropland, and forest) range from a loss of 700 Mha to a gain of 70 Mha by 2050 (IPCC 2019).

Reviews of the scientific literature on LULC modeling have noted that the models employed to produce these types of global projections concentrate their efforts on the representation of agricultural activities without equal attention being devoted to some categories of natural vegetation (Schmitz et al. 2014; Alexander et al. 2017; Prestele et al. 2017). Data quality and availability, together with a long tradition of studies on the influence of market forces on land use and land use change (von Thünen 1966; Chomitz and Gray 1996), might explain why so often the impact of climate change on LULC is investigated through its effects on productivity and prices (Doelman et al. 2018; Molotoks et al. 2020; Zabel et al. 2019). Even when agricultural models are built on sophisticated global vegetation models, LULC change is mainly driven by the future of agriculture: with forest losses almost perfectly mirroring cropland expansion and often the same type of vegetation (for example, tropical forests) persisting even at very high temperatures (Havlík et al. 2018; Schaphoff et al. 2018; Sitch et al. 2003).

However, abundant evidence in the literature indicates that the geographical distribution of all terrestrial ecosystems will be substantially affected by climate change (García Criado et al. 2020; Han et al. 2018; Malhi et al. 2009; Peñuelas et al. 2013; Rees et al. 2020; Ruiz-Pérez and Vico 2020. Studies show that increased temperature and precipitation are key factors influencing grassland vegetation growth (Han et al. 2018; Ziska et al. 2011) and savanna ecosystems (Anadon et al. 2014; Holmgren et al. 2013; Kulmatiski and Beard 2013). Extensive regional declines in forest productivity linked to a changing climate have already been observed in eastern Alaska and western Canada (Beck and Goetz 2011), as well as western central Eurasia and western North America (Buermann et al. 2014).

Of particular concern are the effects of climate change on natural vegetation types that thrive in narrow climatic conditions. For example, closed canopy forest appears to have an upper limit on the temperatures that can support its growth (Wright et al. 2009). Physiological studies support this observation, finding that midday temperatures in the 33–38 °C range depress net photosynthesis in rainforest trees (Pons and Welschen 2003) which can decline by as much as 40–50% as temperatures rise to near 40 °C (Slot and Winter 2018).

Similar to previous studies, our main objective is to use a global scale model to evaluate the risk of incursions of cropland into natural habitats, but we do so while also exploring the implications of the potentially radical reorganization of natural vegetation due to climate change in line with the implications of the ecological literature. The patterns revealed by the results cast the role of cropland expansion in future losses of natural areas in a different light than is usually considered and provoke significant questions about global and local policies intended to protect natural environments.

## Materials and methods

We simulate global changes in future LULC using a model that relies on two distinct components: a natural vegetation and a cropland component. The results of the full model are obtained by first simulating the location of natural vegetation in the absence of crop production and then by allocating land to crop production based on the results of an economic model of the global agricultural economy. The unit of observation is represented by a pixel of half-degree in size (roughly 3000 km^2^ near the equator).

### *Identifying important climatic extremes*

The model is formulated to ensure that it can be responsive to climatic conditions uncommon in the recent past, but which will be much more common in the future.

**Fig. 1:**
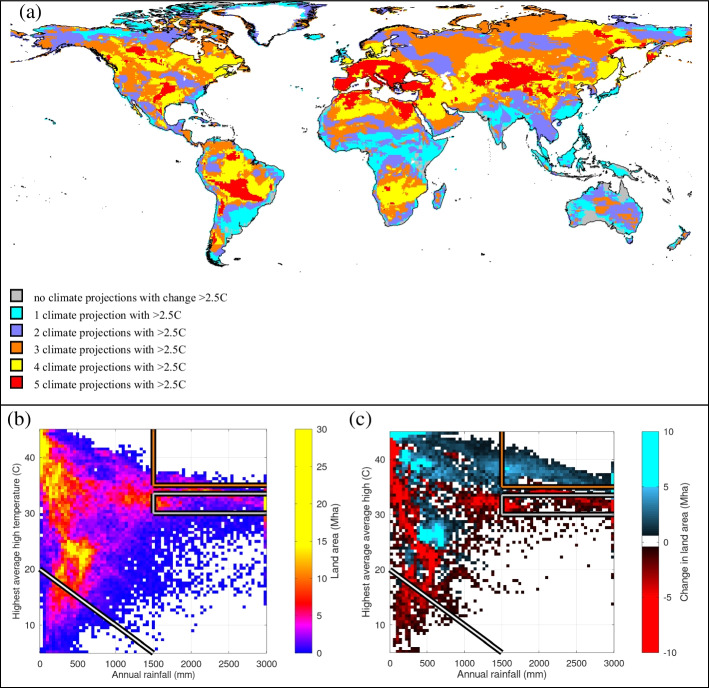
**a** Number of climate models projecting more than 2.5 °C increase in average high temperature during the warmest month. **b** Distribution of land area across temperature and rainfall in 2005. Note that the colors are non-linear to accommodate the extreme values. **c** Change in the land area for each climate combination 2005–2050. Future climates reflect the average of five climate projections (GFDL-ESM4, IPSL-CM6A-LR, MPI-ESM1-2-HR, MRI-ESM2-0, and UKESM1-0) using RCP 8.5. Note that the colors are non-linear to accommodate the extreme values while white represents very small changes or unrepresented combinations

Large areas of natural vegetation will confront a future with very different growing conditions. Comparing the average daily high temperature of the warmest month in 2005 with projections for the year 2050 across five different climate models, we find that at least three of them project a large (> 2.5 °C) increase in temperature on 56% of the land surface (Fig. 1a). The potential for heat stress to interfere with plant growth will increase substantially in the future with significant repercussions for many landscapes.

The potential impact on natural vegetation can be apprehended by comparing panels b and c in Fig. 1. Panel b reports the amount of land area that experienced a particular combination of temperature and precipitation in 2005. Panel c shows, for the same temperature/precipitation combination, how the area under those conditions is expected to change by 2050. For example, the diagonal white line indicates the approximate transition between the cool and dry climates associated with tundra (Körner 1998) and the warmer/wetter climates that support a wider variety of vegetation types. Under the projected climate change, the amount of area exhibiting conditions suitable for tundra decreases dramatically. Climates typically associated with tropical rainforests are indicated by the gray box: temperature between 30 and 33.5 °C with rainfall above 1500 mm per year (Malhi et al. 2009; Wright et al. 2009). Similar to tundra, the amount on land where these climate conditions can be found becomes less common. Warmer “fringe” conditions with temperatures above 35 °C (orange box) are projected to be present on considerably more land than 2005.

If climate projections are correct, we should expect significant spatial reorganization of natural vegetation around new climatic conditions. Importantly, these anticipated changes are independent from the fate of agricultural production.

### Natural vegetation model

The natural vegetation model projects the fraction in each pixel expected to be covered by different natural vegetation types based on the statistical relationship between natural vegetation and climate conditions. While the model parameters are estimated based on real world data, its simulations are independent of initial vegetation type conditions; that is, both the baseline and future vegetation distributions are determined solely by the corresponding climate conditions and the estimated parameters. This combination of pure statistical approach and lack of path dependency is in contrast to most other global models which are strongly influenced by historical vegetation patterns.

The climate data used in the model are based on the Princeton Global Forcing (Sheffield et al. 2006). The starting point in time is meant to be 2005 for which we compute average values over the interval 1995–2015 (instead of using the data from 2005 only). The end-point of the analysis is 2050 which, again, we operationalize as the averages over the interval 2040–2060. Climate data for the future scenarios are drawn from five GCMs (GFDL, IPSL, MIR, MPI, and UKESM) under the representative concentration pathway 8.5 (SSP2/RCP 8.5 for CMIP6). Multidecadal averages are extracted from the GCM data for 2005 (1995–2015) and 2050 (2040–2060). These averages are applied to the Princeton Global Forcing/baseline data so that the baseline and future cases are commensurate with each other and across GCMs (Müller and Robertson 2014). All climate data used by the actual model are averages, not any particular year’s weather.

Data for the location of natural vegetation come from three different sources: MODIS (covering 2001-2012; Channan et al. 2014; Friedl et al. 2010), GLC2000 (targeting 2000; Bartholome and Belward 2005), and GlobCover (targeting 2005, produced by ESA and the ESA GlobCover project led by MEDIAS France/POSTEL; Bicheron et al. 2008). The raw categories in each dataset are consolidated into nine major categories as detailed in Table 1: barren (primarily desert), tundra (both boreal and alpine), shrubland, grassland, savanna, woody savanna, evergreen needleleaf forest (primarily boreal), deciduous/mixed forest, and evergreen broadleaf forest (primarily tropical). Using multiple land datasets closely clustered in time but constructed by different processes helps us establish that the conclusions we draw are not dependent on the idiosyncrasies or peculiarities of a particular dataset.

**Table 1:** Harmonized natural vegetation types and their constituents. Numbers in parentheses indicate the numeric code in the original datasets. Italicized entries indicate categories associated with cropland

Harmonized category	GLC2000	GlobCover	MODIS
Tundra	Latitude north of 58 N or elevation above 4000 m: Herbaceous cover, closed to open (13) Sparse herbaceous or sparse shrub cover (14) OR Latitude north of 48 N or elevation above 4000 m: Shrub cover, closed to open evergreen (broadleaved or needleleaved) (11) Shrub cover, closed to open, deciduous (broadleaved or needleleaved) (12) OR Latitude north of 35 N: Tree cover burnt (mainly boreal forests) (10) Mosaic of tree cover and other natural vegetation (crops possible) (9)	Latitude north of 58 N or elevation above 4000 m: Mosaic forest or shrubland/grassland (110) Closed to open (broadleaved or needleleaved, evergreen or deciduous) Shrubland (130) Sparse vegetation (150) Mosaic grassland/forest or shrubland (120) Closed to open herbaceous vegetation (grassland, savannas or liches/mosses) (140) Bare areas (200) OR Latitude north of 48 N or elevation above 4000 m: Closed to open grassland or woody vegetation on regularly flooded or waterlogged soil—fresh, brackish, or saline water (180)	Latitude north of 48 N: Closed shrublands (6) Open shrublands (7) Woody savannas (8) Savannas (9) Permanent wetlands (11) OR Latitude north of 58 N or south of 58 S: Grasslands (10) OR Elevation above 4000 m: Open shrublands (7) Grasslands (10) Barren/sparse (16)
Evergreen needleleaf forest	Tree cover, needleleaved evergreen, closed to open (4)	Closed needleleaved evergreen forest (70) Open needleleaved deciduous or evergreen forest (90) Closed to open mixed broadleaved and needleleaved forest (100)	Evergreen needleleaf (1)
Evergreen broadleaf forest	Tree cover, broadleaved evergreen, closed to open (1) Tree cover, closed to open, regularly flooded; swamp (7)	Closed to open broadleaved evergreen or semi-deciduous forest (40) Closed to open broadleaved forest regularly flooded (semi-permanently or temporarily)—fresh or brackish water (160)	Evergreen broadleaf (2)
Deciduous/mixed forest	Tree cover, broadleaved deciduous, closed (2) Tree cover, needleleaved deciduous, closed to open (5) Tree cover, mixed leaf type, closed to open (6) Shrub cover, closed to open, evergreen (broadleaved or needleleaved) (11)	Closed broadleaved deciduous forest (50)	Deciduous needleleaf (3) Deciduous broadleaf (4) Mixed forest (5)
Shrubland	No clear analog	NOT already tundra: Mosaic forest or shrubland/grassland (110)	NOT already tundra: Closed shrublands (6) Open shrublands (7)
Woody savanna	NOT already tundra: Tree cover, broadleaved deciduous open (3) Mosaic of tree cover and other natural vegetation (crops possible) (9)	NOT already tundra: Open broadleaved deciduous forest/woodland (60) Closed to open (broadleaved or needleleaved, evergreen or deciduous) shrubland (130) Closed broadleaved forest or shrubland permanently flooded - saline or brackish water (170) Closed to open grassland or woody vegetation on regularly flooded or waterlogged soil—fresh, brackish, or saline water (180)	NOT already tundra: Woody savanna (8)
Savanna	NOT already tundra: Shrub cover, closed to open, evergreen (broadleaved or needleleaved) (12)	No clear analog	NOT already tundra: Savanna (9)
Grassland	NOT already tundra: Herbaceous cover, closed to open (13) Sparse herbaceous or sparse shrub cover (14)	NOT already tundra: Mosaic grassland/forest or shrubland (120) Closed to open herbaceous vegetation (grassland, savannas or lichens/mosses) (140) Sparse vegetation (150)	NOT already tundra: Grasslands (10)
Barren	Bare areas (19)	NOT already tundra: Bare areas (200)	NOT already tundra: Barren/sparse (16)
Excluded (*cropland*)	*Cropland (16)* *Mosaic of cropland/tree cover/other natural vegetation (17)* *Mosaic of cropland/shrub or herbaceous cover (18)* Tree cover, closed to open, flooded; mangrove forest (8) Regularly flooded (15) Water bodies (20) Unnamed but clearly water (23) Urban areas (22) Snow or ice (21)	*Post-flooding or irrigated croplands (or aquatic) (11)* *Rainfed croplands (14)* *Mosaic cropland/vegetation (grassland/shrubland/forest) (20)* *Mosaic vegetation (grassland/shrubland/forest)/cropland (30)* Water bodies (210) No data but usually water (230) Artificial surfaces and associated areas (urban areas) (190) Permanent snow and ice (220)	Water (0) Permanent wetlands (11) (south of 48 N) *Croplands (12)* *Cropland/natural mosaic (14)* Urban/built-up (13) Snow and ice (15)

The spatial resolution of each dataset is coarsened to a common half-degree grid to determine what fraction of each half-degree pixel is occupied by the different vegetation types. In the case of the MODIS dataset, the fractions also represent the average over the 12 years of available data. These fractions constitute the dependent variable of the natural vegetation model.

The econometric model of choice for this type of dependent variable is a fractional multinomial logit which, unlike standard multinomial logit, considers the case where the variable is made of fractions that sum up to one (Mullahy 2015; Murteira and Ramalho 2016).

We employ a piecewise approach in the natural vegetation model. The data domain is divided into four zones: very wet conditions (annual rainfall greater than or equal to 1500 mm) with a distinctive dry season, very wet without a dry season, not very wet (annual rainfall less than 1500 mm) with a dry season, and not very wet without a dry season (Table 2). This allows us to generate parameter estimates that better capture the effects of extremely warm and extremely wet conditions that are likely to be more common and important in the future. While other forms of partitioning are possible, this particular choice was supported by likelihood-ratio tests as well as use of the Schwarz information criterion for model selection.

**Table 2:** Variables used in natural vegetation model with summary statistics. Three variables are directly used as explanatory variables while the other two are used in combination to define the domains of the four sub-models

	Climate 2005 (for estimation: only locations with natural vegetation)	Climate 2005 (for simulation: full land area)	Climate 2050 (for simulation: average across 5 climate models and full land area)	Notes
Explanatory variables				
Average daily high temperature during the hottest month (°C)	Mean 29.9 Stdev 8.40	Mean 29.6 Stdev 8.43	Mean 34.2 Stdev 7.99	Reflects the worst heat stress (in warm climates) or growing opportunities available (in cold climates)
Average daily low temperature during coldest month (C)	Mean −1.8 Stdev 18.8	Mean −0.003 Stdev 18.3	Mean 3.81 Stdev 17.0	Indicates the degree of temperature seasonality and the risk of freezing
Average annual rainfall (mm)	Mean 758 Stdev 748	Mean 847 Stdev 768	Mean 926 Stdev 800	Captures overall moisture availability
Partitioning variables				
Presence of a distinctive dry season (fraction)	Fraction of area with a dry season, 0.316	Fraction of area with a dry season, 0.329	Fraction of area with a dry season, 0.368	Used for partitioning the piecewise model. A dry season is present if the total precipitation across the driest three months is less than 60 mm and across the wettest 3 months is more than 180 mm
Annual rainfall ≥ 1500 mm	Fraction of area with very high rainfall, 0.151	Fraction of area with very high rainfall, 0.173	Fraction of area with very high rainfall, 0.204	Used for partitioning the piecewise model. Very high rainfall situations

Based on the literature that studies the non-anthropogenic drivers of transitions from one natural vegetation type to another, in addition to the two partitioning variables, three explanatory variables are constructed for use in the natural vegetation model (Han et al. 2018; Ziska et al. 2011; Anadon et al. 2014; Holmgren et al. 2013; Kulmatiski and Beard 2013; Wright et al. 2009; Pons and Welschen 2003; Slot and Winter 2018). The variables are summarized in Table 2. Within each partition, the structure of the model is the same: the explanatory variables enter the model specification linearly, quadratically, and with interaction terms. The parameters of the econometric model are estimated using the data from 2005. This allows us to recreate the vegetation patterns from 2005 and then to simulate the distribution of vegetation in 2050 using the various future climates. We use the same model specification on each one of the three land-cover datasets to obtain three sets of parameters and then simulations. The results can be compared and/or averaged with each other to help ensure that our conclusions are not based on the idiosyncrasies of a particular dataset. Hence, we refer to a single natural vegetation model which has three variations, each based on one of the observed land datasets.

### Cropland model

The cropland model geographically allocates the amount of cropland required to satisfy human and animal needs for agricultural products. The amount of cropland is derived from the IMPACT modeling suite (Robinson et al. 2015) based on climate effects from RCP8.5 and using SSP2 drivers for population and income (the settings used for most applications of that model). IMPACT models the behavior of the competitive global agricultural market and simulates supply, demand, and prices for agricultural commodities across 320 sub-national regions called food production units (FPUs) as described in Online Resource 1. We group the cropland area information from IMPACT into five major crops (maize, rice, wheat, sorghum, soybeans, representing about 52% of harvested area; FAOSTAT 2022) and place all other crops together into a sixth composite crop. For all crops, rainfed and irrigated areas are considered separately.

The cropland area is allocated within each FPU across the pixels that are most climatically and economically suitable for agricultural production. The suitability of each pixel for crop production is based on a series of crop-specific “attractiveness indices.” Each attractiveness index is the weighted sum of four components. The first two components are static across time and crop: cost of access to small cities and a measure of how flat the area in the pixel are believed to be. The cost of access measure is based on the time required to travel from the pixel to the most accessible small city (Nelson 2008). All else being equal, closer is better for cropland than farther. The amount of diversity in elevation within a pixel is used to determine how flat the pixel is and it is derived from fine-grained elevation data (GLOBE Task Team et al. 1999). Areas with more uniform elevation are easier to farm than highly varied terrain and are more attractive. The third component is constant across crops but changes through time. Based on the climate data, we consider that the closer future climate conditions are to those historically associated with crop production, the more favorable the pixel will be for cropland. The last component is crop specific, and it is based on the expected yield if that crop were to be grown using the appropriate water source (irrigated or rainfed). These potential yield maps for the five crops considered are generated using the DSSAT crop modeling framework at each location (Hoogenboom et al. 2019a; Hoogenboom et al. 2019b; Jones et al. 2003; Robertson 2017). Higher yields are preferred to lower yields. The allocation process is explained more fully in Online Resource 1.

We assume that, when in demand, cropland will always be carved out of natural vegetation areas. Therefore, within each pixel, the area initially allocated to natural habitats by the natural vegetation model is reduced to accommodate the necessary cropland. This is done by decreasing each vegetation type proportionately and thus retaining their relative proportions. The algorithm ensures that all the cropland area from the IMPACT model is allocated to individual pixels (more details are provided in Online Resource 1).

While the model explicitly tracks the total amount of cropland, this type of comparative static analysis may underestimate the influence of cropland on natural vegetation in some situations such as shifting cultivation or systems incorporating fallow rotations. On the other hand, the simple, equal-opportunity rule for displacing natural vegetation may overestimate its influence on types like forest which are difficult to convert.

Pasture is not explicitly considered in our model for several reasons. First, as a practical matter, pasture as a land type does not appear in the remotely sensed global land data products. Second, as it has already been noted by others (Alexander et al. 2017), defining what constitutes pasture consistently across the globe is prone to disagreement and mistakes. Some pastures are used constantly and intensively while others only intermittently support a low density of animals. As a consequence, there is no globally consistent and viable method to assess the productivity or suitability of a particular location for pasture either absolutely or in relation to cropland. Furthermore, as noted earlier, pasture area is generally thought to have been declining over the last few decades and is expected to continue to do so (Hasegawa et al. 2017; Dietrich et al. 2019; Kreidenweis et al. 2018; Schmitz et al. 2014)**.** Thus, we do not explicitly model pasture and implicitly assume that pasture is contained within natural vegetation types or shares space with cropland.

### Natural vegetation model performance assessment

The inconsistent definitions of natural vegetation types create challenges in assessing model performance.

**Fig. 2:**
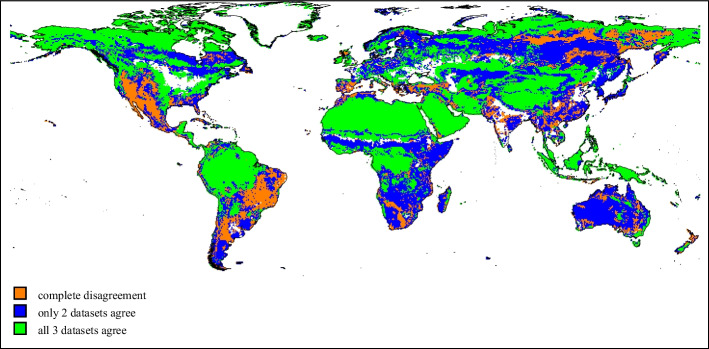
Level of agreement between observed datasets on the predominant land type. Note: white areas indicate locations where all three datasets report only combinations of water, ice/snow, and cropland

First of all, we do not know which dataset more accurately represents the “true” global land cover. The datasets are not in perfect agreement regarding the area covered by land, ice, and water, which leads to slightly different numbers of pixels being available for the model estimation. Furthermore, despite the harmonization of the LULC categories, important differences among the datasets remain. When only the predominant category in each pixel is considered, there is a general agreement regarding the location of evergreen broadleaf forest, barren land, and tundra, but substantial disagreement remains for the other natural vegetation categories as seen in Fig. 2. Substantial differences are also present in the non-dominant categories in each pixel.

In order to perform an assessment that includes all the categories of natural vegetation, we use the concept of Kullback-Leibler divergence, abbreviated as KL (Kullback and Leibler 1951). The KL divergence is a commonly used measure of how far an arbitrary probability distribution *p* is from a reference probability distribution *q*. The measure is not symmetric (i.e., KL(*p*;*q*) ≠ KL(*q*;*p*)), hence the use of the term divergence as opposed to distance. For probability distributions of discrete variables, the KL divergence is the expectation of the difference between the logarithmic probabilities calculated over one of the probability distributions for all possible outcomes “*i*” (Eq. 1).

1 $$\mathrm{KL}\left(p;q\right)=\sum\limits_ip_i\left(\log\;p_i-\log\;q_i\right)$$

The KL divergence is always nonnegative and is equal to zero when the two probability distributions coincide. Treating the land-cover shares reported by the datasets as probabilities and summing across all land types (“*j*”) and locations (i.e., pixels “*l*”), we can perform a pairwise comparison of the datasets and assess how they differ from each other. Calling the land cover-shares *p*_*lj*_ (for type *j* at location *l*) for one dataset and *q*_*lj*_ for the other, the KL divergence between the two datasets is expressed by Eq. 2.

2 $$\mathrm{KL}\left(p;q\right)=\sum\limits_l\sum\limits_jp_{lj}\log p_{lj}-\sum\limits_l\sum\limits_jp_{lj}\log q_{lj}$$

Furthermore, if the distribution *p*_*lj*_ denotes the land-cover shares of a dataset and *q*_*lj*_ the land-cover shares simulated using our natural vegetation model, the second term in the KL divergence is precisely the value of the log-likelihood function of our model since it is estimated using the maximum likelihood method (Bishop 1995). This suggests a method of assessing the performance of the natural vegetation model by directly comparing the KL divergence among the datasets with the KL divergence between the datasets and the shares simulated by the natural vegetation model.

Taking advantage of having multiple datasets and assuming that the “true” land-cover shares always fall between the shares recorded by the datasets, if the observed datasets are farther apart from one another than the model simulations are from the datasets, we gain confidence that the model results are not worse at representing the true land cover than the datasets. In other words, in using the model results, we do not risk making an error that is greater than the one made when choosing one dataset over the others. On the other hand, if the model simulations are farther from the datasets than the datasets are from each other, such confidence vanishes and we consider the model results not worth using.

### Cropland model performance assessment

It is difficult to quantify and accurately locate cropland in the three remotely sensed datasets mostly because they contain mixed categories in which cropland and other types of natural vegetation coexist. For example, the mosaic vegetation category in GlobCover can contain between 20 and 50% of cropland and MODIS’s cropland/natural vegetation mosaic category can contain between 40 and 60% of cropland. Such broad definitions unsurprisingly lead to general agreement between the datasets on the core cropland areas. Our assessment of the performance of the cropland model is based on a pixel-by-pixel comparison between the model’s cropland allocations for the year 2005 and the three datasets. If the model allocates cropland to a pixel in which at least one of the remotely sensed datasets reports the presence of cropland, we consider that prediction to be correct.

## Results

### Model performance

Given the datasets used and the corresponding model simulations, we find that the model performs satisfactorily. The six pairwise KL divergences between the datasets range from 48,203 to 79,866, averaging 66,072.5. On the other hand, the worst KL between an estimated model variation and the dataset used for its estimation is 33,237.7. Thus, the disagreement among the datasets is greater than the disagreement between the model variations’ predictions (based solely on climate) and the datasets. Use of the model results is therefore warranted.

An analysis of the predominant categories in each pixel across datasets also supports the idea that the model simulations more closely resemble their corresponding datasets than the datasets match each other. The datasets agree 50 to 59% of the time, while the model matches the predominant category of the datasets 55 to 66% of the time. Among the land types that are best matched are tundra and evergreen broadleaf forest.

The geographical allocation of cropland appears to be in good agreement with the location of cropland reported by the three datasets. We find that 84.0% of the allocated cropland falls in pixels for which GLC2000 shows at least 10% coverage by a cropland category. The corresponding value for GlobCover is 87.4% and for MODIS, it is 76.5%. Combining them all together, 93.2% of allocated area falls in pixels for which at least one of the remotely sensed datasets shows at least 10% coverage by a cropland category.

### LULC changes at a global scale

We reproduce the LULC patterns of 2005 and project LULC into the future for 2050 in two different ways. One set of simulations includes both cropland and natural vegetation models (i.e., full model) and accounts for the full effects of climate change and socio-economic growth. A second set of simulations uses only the natural vegetation model and simulates a world without cropland to isolate the effects of climate change on natural vegetation.

We report the average of multiple model runs that use five alternative future climate projections and three different land-cover datasets. Averaging the outputs together both simplifies the presentation and helps avoid peculiarities arising from individual datasets (whether climate drivers or natural vegetation observations).

The results of the full model show substantial changes through time when aggregated to the global level. Cropland is expected to expand by 232 million hectares (Mha). Large changes in areas are also projected for some natural vegetation categories. Specifically, grassland and deciduous mixed forest are expected to grow by 474 Mha and 118 Mha, respectively. In contrast, evergreen broadleaf forest and tundra are projected to decrease by 180 Mha and 608 Mha. The remaining categories each exhibit changes of less than 80 Mha. These changes are the product of the compounded effects of a greater demand for agricultural products by a larger and richer human population, the climate change impacts on crop productivity, and geographical shifts in the suitability of land for natural vegetation types.

**Fig. 3:**
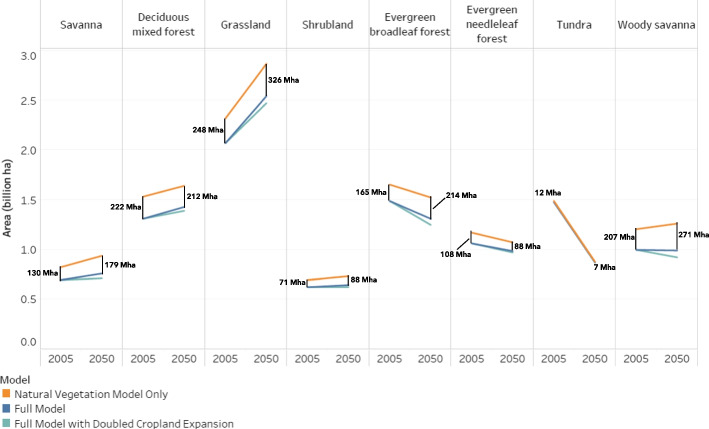
Evolution of global area by natural vegetation type. Values (in billions of hectares) are the average across all climate scenarios and natural vegetation model variations. Gaps between the upper/orange and middle/blue lines represent the amount of natural vegetation displaced by cropland. The lowest lines reflect the scenario wherein cropland expansion is double that of the realistic case

A comparison between the results of the full model and the natural vegetation model allows us to assess the impact of cropland expansion on natural environments (Fig. 3). Most of the projected changes in area are qualitatively similar: that is, the lines in each pair slope in the same direction. This suggests that cropland expansion either compounds with or is not able to offset the trends imposed by climate change. The only exception is woody savanna for which more suitable area is made available under future climates, but it is expected to decrease in area because of cropland expansion into those same areas.

Other global models have projected both more cropland expansion than the amount used here (Hasegawa et al. 2017; Dietrich et al. 2019; Kreidenweis et al. 2018; Schmitz et al. 2014). To address this range of possibilities, we developed a speculative scenario in which the additional cropland in each region and for each crop was doubled from the IMPACT projections and no crops were allowed to contract. Specifically, instead of an additional 232 Mha, this scenario allocated 573 Mha more than the 2005 level. Figure 3 shows that even with double the growth in cropland, the patterns are mostly unchanged.

The results provide an opportunity to analyze how cropland displaces individual natural vegetation types. For example, in 2005, cropland claims 108 Mha that would “naturally” be covered by evergreen needleleaf forest. In 2050, this amount decreases by 21 Mha to a total of 88 Mha. (Rounding causes slight discrepancies in the reported arithmetic.) The decline is not sufficient to offset the losses driven by changing climates: after cropland is considered, evergreen needleleaf forest shrinks by 77 Mha. Savanna moves in the opposite direction: the simulations indicate that in 2050, there will be an additional 65 Mha even though cropland will be eroding 49 Mha more than it did in 2005. In contrast, the total area covered by woody savanna is projected to decrease because cropland takes an additional 63 Mha compared to 2005 and this amount offsets the growth in area caused by climate change (+ 57 Mha).

The impact of climate change dwarfs that of cropland expansion for tundra and grassland, and it is substantially greater in the case of evergreen broadleaf forest. Cropland is almost absent from tundra and is projected to have even less conflict with tundra in the future. We observe the opposite situation for grassland: large and expanding conflicts with cropland which are much more than offset by new areas having favorable climates in the future. In the case of evergreen broadleaf forest, the effects of climate and cropland expansion reinforce each other, but climate is the main driver. Climate change reduces suitable evergreen broadleaf forest environments by 130 Mha and cropland expansion encroaches on an additional 50 Mha of forest compared to 2005.

It is worth noting that the patterns of cropland encroachment into natural vegetation depend on the “migration” of vegetation to more suitable areas. In fact, without considering the effects of climate change on natural vegetation, the impact of cropland expansion would be different. For example, with stationary evergreen broadleaf forests, cropland encroaches into some 56 Mha by 2050 instead of the 50 Mha we found deploying our full model.

### Local versus global effects

**Fig. 4:**
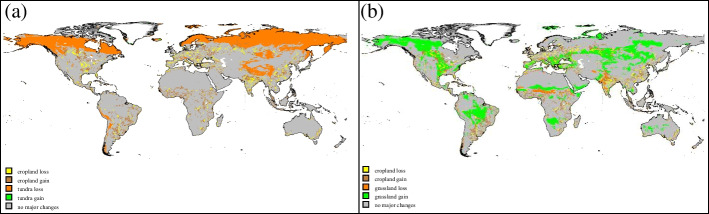
Maps of pixels experiencing changes of at least 10% of their area. **a** Tundra. **b** Grassland. Pixels whose cropland changes by at least 10% of the pixel area are overlaid, sometimes obscuring the natural vegetation changes

Results at the global level provide valuable context, but do not reveal local changes driven by shifting land suitability due to climate change and local expansions (or contractions) of cropland. For tundra (Fig. 4a), climate change leads to a rather uniform area contraction with cropland playing almost no role. Results for grassland instead (Fig. 4b) show zones of both expansion and contraction mixed with regions where cropland encroaches more and others where cropland retreats. Assessing these patterns is of particular importance in places where the natural vegetation type is of high ecological significance or underpins the livelihoods of the people living in that area (Egoh et al. 2012). To illuminate this point, we focus on evergreen broadleaf forests (which are primarily tropical rainforests) because of their richness in biodiversity, cultural importance, and large carbon stocks (Baccini et al. 2017).

**Fig. 5:**
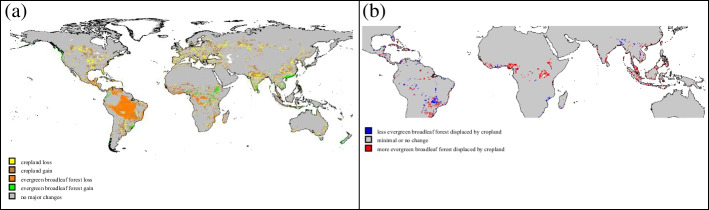
Maps of changes related to evergreen broadleaf forest. **a** Changes in the evergreen broadleaf forest share of each pixel between 2005 and the average of the climate projections for 2050 that are greater than 10%. **b** Type of change for amount of evergreen broadleaf forest displaced by cropland between the same time periods

Climate conditions are projected to be less favorable for evergreen broadleaf forest almost everywhere. Mountains may offer a limited escape route but poleward migration within the tropics is not viable because all bordering land areas are warmer and drier. Essentially, unlike other vegetation types, there will be no refuges for evergreen broadleaf forest to escape to (much like other research has found (Wright et al. 2009)). The implications of these changes according to our model are shown in Fig. 5a: the very few locations that show any potential for increases in area covered by evergreen broadleaf forest are on mountains at the margins of already existing rainforest areas.

Though evergreen broadleaf forest losses are nearly universal, the relationship to cropland differs by region. The four major concentrations of tropical forest we look at here are Southeast Asia/Indonesia/Papua New Guinea (representing 20–25% of the world’s evergreen broadleaf forests), the Guinean forests of West Africa (about 5%), the Amazon forest in South America (about 40%), and the Congolian forests of Central Africa (about 10%).

In the tropical forests of Southeast Asia, Indonesia, and Papua New Guinea, climate change may increase the potential area for evergreen broadleaf forest driven by climate change by 16.5 Mha. However, cropland expansion will be even greater, 27.2 Mha. The result is that the conflict between cropland and forest in 2050 will increase to about 27% of the total potential forest area, up from the 21% in 2005. Since cropland expansion hinders the forest while climate change helps, the relative contributions are each larger than 100%: cropland expansion is responsible for 254% of the overall loss of 10.7 Mha which is offset by the negative 154% due to climate change.

The smaller Guinean forests of West Africa also start 2005 with a high rate of tropical forest displaced by cropland (20%). By 2050, they will experience a loss in potential forest area made worse by additional pressure from cropland that encroaches even more on the dwindling suitable area for forest (red/“more” pixels in Fig. 5b). This will lead to 34% of potential forest area displaced by cropland. Of the 11.5 Mha decline in forest area, 27% is driven by climate and 73% by additional conflict with cropland.

The Amazonian and Congolian rainforests have much lower fractions displaced by cropland. By 2050, the simulations show the Amazonian forest of South America with 2.6% of its evergreen broadleaf forest displaced by cropland. Similarly, the Congolian rainforest of Central Africa is simulated to have 5.6% of its potential area displaced by cropland in 2050. Although climate is the main driver of loss in both regions, the magnitudes are different.

The Amazonian forests of South America will be the hardest hit by climate, as is clearly shown in Fig. 5a: by 2050, the projected reduction is 111.8 Mha (17% of the original area). This contraction of tropical forest will be almost entirely driven by climate as the amount of forest area displaced by cropland in 2050 increases by only 1.4 Mha (from 13.3 to 14.7 Mha). The forest will be retreating along with some cropland while other cropland expands. This is shown by the red/“more displacement” in the periphery and presence of blue/“less displacement” pixels in the core of the Amazon seen in Fig. 5b. In this region, the effect of climate dominates that of cropland: 99% of the net losses of evergreen broadleaf forest are due to climate and only 1% to cropland expansion.

The impacts of climate are also dire in the Congolian forests of Central Africa. By 2050, 12.1 Mha of evergreen broadleaf forest will be lost, or 8% of the original area. Of that, only 3.1 Mha (about 26%) comes from additional cropland incursion and the remaining 9.0 Mha (about 74%) is attributable to climate.

## Discussion

Our analysis shows that by accounting for a possible radical reorganization of natural vegetation, new perspectives are revealed concerning the role played by agricultural production in displacing natural vegetation. The modeling results show that the magnitude of the changes in natural vegetation due to climate change could greatly outweigh the effects of cropland expansion. Furthermore, the results reveal that effective protection of valuable natural areas will depend on understanding the complex patterns of expansion and contraction driven by a combination of changes in climate, market forces, and progress in agricultural technology.

Anthropogenic climate change will be habitat destruction at the ecosystem level for tropical rain forest and tundra. The area covered by tropical rain forests could decline due to deteriorating climatic conditions independently of cropland encroachment and despite legal or physical protections (a conclusion consistent with other research; IPCC 2019; Elsen et al. 2020; Lawler et al. 2020). As for tundra, the melting of the permafrost and higher temperatures in alpine contexts could radically change which species can survive in those areas. The disappearance of tundra, which has a potentially significant impact on the release of methane (Turetsky et al. 2019), is unrelated to cropland expansion but would still have important knock-on effects. For both tropical rain forest and tundra, there will be no significant new refuges with favorable climates to escape to.

In analyzing the role of cropland in the loss of natural habitats, localized contexts matter. Each natural vegetation type has its own response to climate change. Cropland does not respond to climate change and market forces in a homogenous fashion nor in concert with neighboring ecosystems. For example, preservation of the Amazonian forests as well as the Congolian forests appears to depend first on stabilizing the climate and only after the climate threat is under control does it depend on the evolution of crop production. Conversely, forest protection can mitigate some of the losses in areas that remain fully or partially climatically suitable in the future. For instance, rainforests in Southeast Asia, especially, along with the Guinean zone of West Africa face strong pressure from cropland expansion. For those areas where forests seem to be able to survive the projected climate change of the next few decades, additional resources should be devoted to understanding how crop production evolves and efforts to protect forests from agricultural expansion should be renewed. Analogous considerations can be made for the other types of natural vegetation that are similarly characterized by complex patterns of contraction and expansion.

Initiatives and policies intending to preserve ecosystems or support livelihoods dependent on the existence of a particular natural environment must account for long-term changes that can determine the outcomes of protection efforts. In essence, the impact of climate change can make conservation efforts either more significant or increasingly futile.

While our projections of cropland expansion are comparable to others, projected changes in areas covered by natural vegetation go beyond what is in the existing literature. This is due to several of the modeling choices made to explore the implications of a radical rearrangement of LULC. Importantly, we chose to use RCP 8.5 as a scenario for future greenhouse gas (GHG) atmospheric concentrations (Riahi et al. 2011). The RCP 8.5 scenario is often considered a “worst-case scenario” for the future climate, and therefore, if there were a considerable reduction in GHG emissions, our modeling results would overestimate temperature and precipitation changes. It should be noted, however, that RCP 8.5 is still the best match for historical CO_2_ emissions, and it is considered the most reliable projection for emissions to mid-century under current policies (Schwalm et al. 2020).

Furthermore, we decided to make the natural vegetation model depend exclusively on climate variables which means that the effects of climate are not mitigated by other factors that affect plant growth (e.g., soil, topography, solar radiation, carbon dioxide fertilization). Research into the interaction between the biosphere and the fast-changing climate is advancing (Christmas et al. 2016; Scheiter et al. 2020), but far from settled. This is mainly because the unprecedented rates of change in CO_2_ concentration (Foster et al. 2017) leave us with no past analog from which to infer the timing and the more nuanced responses of vegetation to the ongoing climatic changes (Thomas et al. 2004). Therefore, we decided to center our analysis on shifts in temperature and precipitation.

The direct effect of elevated carbon dioxide levels is missing from our model. Due to the lack of meaningful variation within the datasets, it cannot be estimated within the cross-sectional approach. It is difficult to determine how this might bias the results. While most or all vegetation will likely benefit from higher carbon dioxide levels, it is the relative benefit under particular conditions which will influence which vegetation types will be able to crowd out others. Whether the fertilization effect will have a large enough differential effect to dominate the influence of temperature and rainfall remains to be seen. And so, again, our model should be viewed as a complement to other models which do include mechanisms relating to carbon dioxide.

The mismatch between rates of change in climatic conditions and the time required by natural vegetation to evolve and adapt to new conditions or seek out new locations implies that vegetation will not quickly find an equilibrium state with the surrounding environment (Peñuelas et al. 2013; Svenning and Sandel 2013). Abandonment due to poor conditions may occur more quickly than colonization by new vegetation, especially for long-lived plants like trees. This also applies to cropland and the disturbance it creates as it expands or moves through the landscape. Hence, we recognize that the comparative static nature of our analysis means that the results of the natural vegetation model do not capture the potential for long-lasting disequilibria nor the complexities of plant competition that will determine new equilibria. However, it provides a clear, if coarse, overview of the pressures for change and the possible futures for natural vegetation.

The results suggest that the effect of climate change on the amount of area suitable for tropical rain forest is so much larger than the effect of cropland incursion that even if all the shortcomings of the model compounded each other, the dominance of climate change would likely persist. At the global scale, the expansion of cropland would have to be roughly double that projected by IMPACT to be equivalent to the effect of climate change. Acknowledging these limitations and calling for more research to overcome them, we note that our results point to a potential miscalculation in the existing assessments of the future impact of cropland on natural vegetation areas.

Our results imply that, by far, the best form of protection for most natural vegetation types is to avoid as much climate change as possible. Still, agriculture can contribute to the effective reduction of greenhouse gas emissions with investments in food production, storage, and distribution systems that favor the storage of carbon in soils and trees and promote the use of practices and technologies that reduce emissions (De Pinto et al. 2020). In this sense, avoiding further cropland expansion into tropical forests and other ecosystems (even in areas that are expected to become less suitable for the ecosystem to be protected) is a valuable effort in so far as it contributes to reducing GHG emissions and preserving carbon stocks.

Overall, it is clear that generalized concerns about cropland encroachment in natural areas must be revisited in favor of more localized analyses that more fully account for the effects of climate change. It appears, therefore, that a new wave of research is necessary to recontextualize the role of agriculture in habitat destruction over multidecadal timescales and understand the pros and cons of increasing agricultural production in a world of shifting ecological (dis-)equilibria driven by climate change.

## Supplementary information

ESM 1 (DOCX 24 kb)

## Data Availability

The datasets generated during the current study will be made available on Dataverse.
