# Supplementary material for: Assessing the future global distribution of land ecosystems as determined by climate change and cropland incursion
Source: Clim Change. 2023 Jul 28;176(8):108. doi: 10.1007/s10584-023-03584-3 (PMC10382346; doi:10.1007/s10584-023-03584-3)
Supplement: Supplementary file 1 — (DOCX 24 kb) [file 10584_2023_3584_MOESM1_ESM.docx]

Supplementary Materials for

Assessing the future global distribution of land ecosystems as determined by climate change and cropland incursion

S1. IMPACT model

The amount of cropland is determined by the IMPACT model which is more formally known as the “International Model for Policy Analysis of Agricultural Commodities and Trade”. A brief description of the model is provided below. A full description can be found in its main documentation (Robinson et al 2015). Similar detail descriptions can also be found in Mason-D’croz et al (2019) and Mason-D’Croz et al (2020).

IMPACT is a partial-equilibrium economic model that simulates national and global markets of agricultural production, demand, and trade associated with 62 agricultural commodities across 159 countries. It combines population and income projections with data based on changes in temperature and precipitation from climate models (simulated yield data from crop models, estimates of water availability from water models). Its outputs reflect the interaction of both biophysical and economic factors.

Climate mediated changes in yields come from two sources: direct effects on yields and indirectly through water availability are captured through linked water models (Mueller and Robertson, 2014). The IMPACT model gives and receives feedback from three water models (global hydrologic model, water basin management model, and water allocation and stress model). The inter-linkage can be configured to reflect the impact of climate change or policy decisions on hydrology or water allocations, thereby allowing simulation of changes in water availability for irrigation and the consequent effects on agricultural production. Agricultural production is specified by models of land supply, and by allocation of land (irrigated and rainfed) to crops. The yield portion of production is influenced by information on yield responses from crop simulation models. Production is modelled at sub-national level (typically aligned with river basins), across 320 regions called “food production units” or FPUs.

The main drivers of the baseline suite of IMPACT scenarios (i.e. Business as Usual scenarios) are gross domestic product (GDP), population, and intrinsic agricultural productivity growth. GDP growth is obtained from the OECD (Dellink et al 2017) and population growth from IIASA (2013). The choices of GDP and population growth are made to allow the IMPACT model to reproduce the Shared Socioeconomic Pathways (SSP scenarios) adopted by the Intergovernmental Panel on Climate Change Fifth Assessment Report (IPCC AR5). The intrinsic yield growth rates are based on past trends and expert opinion. Details of all the input data to the IMPACT model, the equations at the core of the model, and relevant citations can be found in the IMPACT documentation (Robinson et al 2015).

IMPACT has a long record of applications and it has been employed in a wide range of analyses, from assessing the potential effects of climate change on global food production and nutrition (Nelson et al 2010; Springmann et al 2016), to explore linkages between agriculture production and food security at the national and regional levels (Hachigona et al 2013; Jallow et al 2013; Sulser et al 2011; Waithaka et al, 2013), to interdisciplinary assessment of economic models (Nelson et al 2014; Wiebe et al 2015) to evaluating the global effects of biofuels production (Rosegrant 2008), to the assessment of economic effects of alternative mitigation policies (De Pinto et al 2016) and the global simulation of technology adoption (Rosegrant et al 2014) to the future of dietary diversity (Mason-D’Croz et al 2019) or the global consequences of animal disease outbreaks (Mason-D’Croz et al 2020).

S2. Cropland allocation algorithm

The cropland portion of the model allocates the total amount of cropland as determined by the IMPACT model to particular locations. While IMPACT provides information about cropland at the FPU level, the algorithm allocates that cropland into some of the half-degree pixels within the FPU. Each pixel is roughly 3000 square kilometers near the equator. The algorithm considers five crops explicitly (rice, wheat, maize, sorghum, and soybeans) and groups all the remaining crops into an “all others” category. IMPACT implements a distinction between irrigated and rainfed productions systems and the algorithm retains that division which results in a total of 12 different crops (6 crops by 2 water systems) that can potentially be allocated to each pixel. The simulated global distribution of cropland is built up by bringing all the FPUs together on worldwide maps.

In the real world, the observed distribution of crops has two important characteristics. Cropland is spread widely across the globe. Most major crops, such as wheat, are grown in many locations, some of which may not seem to be particularly favorable. Secondly, a mixture of crops is usually observed, even at small scales. Reproducing these features, at least in a broad sense, is the task of the allocation algorithm.

While the precise mechanism used to allocate cropland is not always readily apparent in the major global models of land use, a common theme is to initialize the allocation process with a known map of cropland and then modify it through time based on costs of conversion and potential yield/profits (for example, Havlík et al 2018; Dietrich et al 2019). This is a useful and powerful approach, but there are potential drawbacks. Pure yield or profit optimization approaches tend to concentrate cropland into narrow zones and segregate crops in unrealistic ways. Thus, starting from a known map helps to maintain realistic cropland patterns in projections. On the other hand, when the initial conditions are emphasized, the potential evolution of cropland is heavily constrained by the geographic patterns observed in the past. We consider this particularly problematic when dealing with climate change since the conditions of the future will be substantially different from those experienced in the past.

The cropland allocation algorithm in this study employs a different set of approaches to deal with the same challenges. It does not use historical distributions of cropland as a starting point which is then modified through time. Rather, both the starting and ending situations are simulated based on the appropriate climates and other attributes of the land; that is, there is no map of cropland distribution used to initialize the process. The prioritization of locations for cropland is guided by an assessment of how “attractive” each pixel is for each crop (see Section S2.1). The algorithm respects three constraints to limit the tendency of the prioritization toward concentration of cropland in any particular pixel and to limit the dominance of a single crop within any particular pixel (Sections S2.2 and S2.3).

S2.1 Attractiveness indices

Each crop has a corresponding “attractiveness index” that is computed for each pixel. The index consists of the weighted sum of four sub-indices, each of which ranges from zero to one. The first one is based on a cost-of-access measure which ranks the pixels based on their distance from major urban areas (Nelson 2008). We use the same value for the starting (2005) and ending (2050) time periods in the simulation, while recognizing the limitation that new cities and markets may develop in the future. The second sub-index ranks pixels by elevation diversity, that is, the amount of variation of elevation (GLOBE Task Team 1999) inside each pixel. Flat pixels (lower elevation diversity) are considered easier to farm than those with more varied terrain. The third sub-index considers the similarity of the pixel’s climate (historical for 2005 and projected for 2050) to climates that are associated with cropland. This similarity is based on the specific combinations of historical temperature and precipitation found in the pixel as compared to those typical of five major cropland regions (two sites in West Africa representing the semi-arid zone and the humid zone, one from India for generally hot conditions, one from the North American Midwest for moderately warm and wet, and one from Ukraine for a cooler and drier temperate climate). The final sub-index is crop specific and is based on the potential yield of that crop under the pixel specific climate for each time period. The potential yield is determined using the DSSAT process-based crop simulation modeling framework (Hoogenboom et al 2019a; Hoogenboom et al 2019b; Jones et al 2003; Robertson 2017).

S2.2 Allocation procedure

Within each FPU, the algorithm proceeds as follows. For each crop, there is a total amount of area that needs to be allocated (as obtained from IMPACT projections). As the allocation proceeds, area in the FPU is moved from the “IMPACT totals” to the individual pixels. At the end of the process, there is no area left to assign and the area in the pixels sum up to the total original crop area indicated by IMPACT.

The crop are put into an arbitrary order. We chose the following sequence: wheat, maize, sorghum, soybeans, rice, and all others. We observed that the order chosen does not significantly affect the final results. Starting with the first crop (i.e., wheat), the algorithm looks for the pixel that has the highest attractiveness index value for that crop. A small amount of area from the total indicated by IMPACT is then allocated to that particular crop (wheat) in that particular pixel. The algorithm cycles through each crop, looking for the most attractive pixels that still have area available for cropland until it has allocated all the area for all crops to the pixels.

Irrigated and rainfed areas are allocated separately. Irrigated areas are fully allocated first. Then rainfed cropland is allocated to the remaining area.

S2.3 Rules and constraints

Other rules are imposed to ensure that cropland area is spread out as well as to encourage crop diversity inside each pixel.

To reduce the concentration of cropland in a few “highly attractive” pixels within each FPU, we limit the amount of area available for cropland within each pixel. Irrigated land is initially confined to how much area is equipped for irrigation defined by the Global Map of Irrigation Areas (Siebert et al 2013) while rainfed cropland can occupy any area not already allocated to irrigated cropland. However, we reduce this physical potential area by setting an arbitrary cap on the percentage of area that is actually made available to the allocator. For irrigated cropland, we initially make only 90%of the area equipped for allocation available for allocation. For rainfed cropland, we set the initial cap at 67% of the physical area (net of the irrigated areas) to force the selection of pixels over a broader region. Higher caps allow greater concentration while lower caps force cropland onto a broader footprint. The particular values were chosen after experimentation with a series of alternative values to strike a balance between concentration, dispersion, and historical cropland distributions as interpreted by SPAM (IFPRI IIASA 2016).

In the cases where the available cropland area at the pixel level is not enough to accommodate th cropland area required by IMPACT, we make some adjustments. When the problem arises with irrigated area, we first relax the 90% area constraint to find more area. If this is insufficient (that is, all the area thought to be equipped for irrigation is already taken), we look at pixels already having irrigation and claim a fraction of the remaining “non-irrigated” area and convert it to irrigated area. The motivating assumption is that it would generally be easier to expand near existing irrigation systems than to build entirely new systems in entirely new locations. In the event these adjustments are not sufficient and more irrigated area needs to be allocated, a fraction of every remaining pixel is claimed until enough area is found. In the extreme, if all the area of the pixels with an FPU is used and it is still not sufficient to satisfy IMPACT’s requirement, we simply reduce the total amount of area to be allocated to match what is available. Future work will create a feedback mechanism to inform IMPACT that it has come up against hard constraints.

The same process is followed for rainfed cropland. First, any unused land equipped for irrigation (after all the irrigated cropland has been allocated) is returned for possible rainfed use. When there is insufficient area at the pixel level to satisfy IMPACT’s requirements, the constraint is raised from 67% to whatever is needed to provide enough area. In the extreme, the full physical area of every pixel can be used and the area to be allocated is reduced to match what is physically possible.

To assist in achieving a sufficient degree of crop diversity within a single pixel, we only allocate a portion of any pixel during any single step of the allocation process cycle. In particular, we assign up to 10% of a pixel’s originally available area in each step, meaning that it takes at least 10 rounds for all of its available area to be allocated.

To discourage a single crop from occupying the entirety of an individual pixel, we enforce two more rules. These rules only apply when there is more than one crop with area remaining to be allocated.

The first is a relative restriction. When searching for the most attractive pixel, beyond requiring area to be available in the pixel, the algorithm looks at the crops already allocated to each pixel. Suppose the crop being allocated is wheat. If wheat already occupies more than 65% of the cropland area already allocated within the pixel, the pixel is not eligible to receive more wheat and the algorithm continues searching for another pixel. As the allocation continues, eventually another crop may allocate some area to that pixel reducing the relative wheat share to a value below 65%. Then, the pixel will again be eligible to receive some wheat area. When there is only a single crop with area left to allocate, this rule no longer applies and the most attractive pixels will be filled in.

The second rule is an absolute restriction: no crop is allowed to occupy more than 50% of the available cropland area of a pixel (regardless of how much has been allocated to cropland) unless it is the only crop with area left to be allocated. Due to the “relative restriction”, this will only apply when the pixel is almost completely allocated. For example, consider a situation where a pixel has 10 ha that can be used for cropland. After a few rounds of allocation, the pixel has 5 ha of wheat and 3 ha of soybeans. In relative terms, this is 62.5% wheat and 37.5% soybeans, so by the relative rule, more wheat can be assigned. However, in absolute terms, 50% is wheat, 30% is soybeans, and 20% is not yet cropland. The absolute restriction means that no more wheat can be allocated to this pixel unless wheat is the only crop with area remaining to be allocated.

References

De Pinto A, Li M, Haruna A, Hyman GG, Martinez MA, Creamer B, Kwon HY, Garcia JB, Tapasco J, Martinez JD (2016) Low emission development strategies in agriculture. An agriculture, forestry, and other land uses (AFOLU) perspective. World Development 87:180-203. https://doi.org/10.1016/j.worlddev.2016.06.013

Dellink R, Chateau J, Lanzi E, Magné, B (2017) Long-term economic growth projections in the Shared Socioeconomic Pathways. Glob. Environ. Chang. 42:200–214. https://doi.org/10.1016/j.gloenvcha.2015.06.004

Dietrich JP, Bodirsky BL, Humpenöder F, Weindl I, Stevanović M, Karstens K, Kreidenweis U, Wang X, Mishra A, Klein D, Ambrósio G. (2019) MAgPIE 4–a modular open-source framework for modeling global land systems. Geoscientific Model Development 12:1299-317. https://doi.org/10.5194/gmd-12-1299-2019

GLOBE Task Team and others (1999) The Global Land One-kilometer Base Elevation (GLOBE) Digital Elevation Model, Version 1.0. Hastings DA, Dunbar PK, Elphingstone GM, Bootz M, Murakami H, Maruyama H, Masaharu H, Holland P, Payne J, Bryant NA, Logan TL, Muller J-P, Schreier G, MacDonald JS (eds). National Oceanic and Atmospheric Administration, National Geophysical Data Center, Boulder, CO, U.S.A. Digital data base on the World Wide Web (URL: http://www.ngdc.noaa.gov/mgg/topo/globe.html, 1999)

Hachigonta S, Nelson GC, Thomas TS, Sibanda LM (eds.) (2013) Southern African Agriculture and Climate Change: A Comprehensive Analysis (Vol. 3). International Food Policy Research Institute.

Havlík P, Valin H, Mosnier A, Frank S, Lauri P, Leclère D, Palazzo A, Batka M, Boere E, Brouwer A, Deppermann A (2018) GLOBIOM documentation. International Institute for Applied Systems Analysis, Laxenburg, Austria. https://iiasa.github.io/GLOBIOM/GLOBIOM_Documentation_20180604.pdf

Hoogenboom G, Porter CH, Boote KJ, Shelia V, Wilkens PW, Singh U, White JW, Asseng S, Lizaso JI, Moreno LP, Pavan W, Ogoshi R, Hunt LA, Tsuji GY, Jones JW (2019) “The DSSAT crop modeling ecosystem” in Advances in Crop Modeling for a Sustainable Agriculture pp. 173-216. Boote KJ (ed.) Burleigh Dodds Science Publishing, Cambridge, United Kingdom

Hoogenboom G, Porter CH, Shelia V, Boote KJ, Singh U, White JW, Hunt LA, Ogoshi R, Lizaso JI, Koo J, Asseng S, Singels A, Moreno LP, Jones JW (2019) Decision Support System for Agrotechnology Transfer (DSSAT) Version 4.7.5. DSSAT Foundation, Gainesville, Florida, USA, https://DSSAT.net

International Food Policy Research Institute (IFPRI); International Institute for Applied Systems Analysis (IIASA) (2016) Global Spatially-Disaggregated Crop Production Statistics Data for 2005 Version 3.2. https://doi.org/10.7910/DVN/DHXBJX, Harvard Dataverse, V9

International Institute for Applied Systems Analysis (IIASA) (2013) SSP Database

Jalloh A, Nelson GC, Thomas TS, Zougmoré R, Roy-Macauley H. (eds.) (2013) West African Agriculture and Climate Change: A Comprehensive Analysis. International Food Policy Research Institute.

Jones JW, Hoogenboom G, Porter CH, Boote KJ, Batchelor WD, Hunt LA, Wilkens PW, Singh U, Gijsman AJ, Ritchie JT (2003) DSSAT Cropping System Model. European Journal of Agronomy 18:235-265. https://doi.org/10.1016/S1161-0301(02)00107-7

Mason-D'Croz D, Bogard JR, Sulser TB, Cenacchi N, Dunston S, Herrero M, Wiebe KD (2019) Gaps between fruit and vegetable production, demand, and recommended consumption at global and national levels: an integrated modelling study. Lancet Planetary Health 3:e318-e329. https://doi.org/10.1016/S2542-5196(19)30095-6

Mason-D’Croz, D, Bogard JR, Herrero M, Robinson S, Sulser TB, Wiebe KD, Willenbockel D, Godfray HCJ (2020). Modelling the global economic consequences of a major African swine fever outbreak in China. Nature Food 1:221–228. https://doi.org/10.1038/s43016-020-0057-2

Muller C, Robertson, RD (2014) Projecting future crop productivity for global economic modeling. Agric. Econ. 45:37–50. https://doi.org/10.1111/agec.12088

Nelson A (2008) Estimated travel time to the nearest city of 50,000 or more people in year 2000. Global Environment Monitoring Unit - Joint Research Centre of the European Commission, Ispra, Italy. Available at http://forobs.jrc.ec.europa.eu/products/gam/

Nelson GC, Rosegrant MW, Palazzo A, Gray I, Ingersoll C, Robertson R, Tokgoz S, Zhu T, Sulser TB, Ringler C, Msangi S, You L (2010) Food Security, Farming, and Climate Change to 2050: scenarios, results, policy options. International Food Policy Research Institute, Washington, DC

Nelson GC, Van Der Mensbrugghe D, Ahammad H, Blanc E, Calvin K, Hasegawa T, Havlik P, Heyhoe E, Kyle P, Lotze‐Campen H, von Lampe M (2014) Agriculture and climate change in global scenarios: why don't the models agree. Agricultural Economics 45:85-101. https://doi.org/10.1111/agec.12091

Robertson RD (2017) Mink: Details of a global gridded crop modeling system. (International Food Policy Research Institute, Washington, D.C. http://ebrary.ifpri.org/cdm/ref/collection/p15738coll2/id/131406

Robinson S, Mason d'Croz D, Islam S, Sulser TB, Robertson RD, Zhu T, Gueneau A, Pitois G, Rosegrant MW (2015) The International Model for Policy Analysis of Agricultural Commodities and Trade (IMPACT): Model description for version 3. IFPRI Discussion Paper 1483. International Food Policy Research Institute, Washington, DC. http://ebrary.ifpri.org/cdm/ref/collection/p15738coll2/id/129825

Rosegrant MW (2008) Biofuels and Grain Prices: Impacts and Policy Responses. International Food Policy Research Institute, Washington, DC. https://ebrary.ifpri.org/digital/collection/p15738coll2/id/10350

Rosegrant MW, Koo J, Cenacchi N, Ringler C, Robertson RD, Fisher M, Cox CM, Garrett K, Perez ND, Sabbagh P (2014) Food security in a world of natural resource scarcity: The role of agricultural technologies. International Food Policy Research Institute, Washington, DC. https://ebrary.ifpri.org/digital/collection/p15738coll2/id/128022

Siebert S, Henrich V, Frenken K, Burke J (2013) Global Map of Irrigation Areas version 5. Rheinische Friedrich-Wilhelms-University, Bonn, Germany / Food and Agriculture Organization of the United Nations, Rome, Italy

Springmann M, Mason-D’Croz D, Robinson S, Garnett T, Godfray HCJ, Gollin D., Rayner M, Ballon P, Scarborough P (2016) Global and regional health effects of future food production under climate change: A modelling study. The Lancet 387:1937-1946. https://doi.org/10.1016/S0140-6736(15)01156-3

Sulser TB, Nestorova B, Rosegrant MW, van Rheenen T (2011) The future role of agriculture in the Arab region’s food security. Food Security 3:S23–S48. https://doi.org/10.1007/s12571-010-0100-5

Waithaka M., Nelson GC, Thomas TS, Kyotalimye, M (eds.) (2013) East African Agriculture and Climate Change: A Comprehensive Analysis. International Food Policy Research Institute.

Wiebe K, Lotze-Campen H, Sands R, Tabeau A, van der Mensbrugghe D, Biewald A, Bodirsky B, Islam S, Kavallari A, Mason-D’Croz D, Müller C (2015) Climate change impacts on agriculture in 2050 under a range of plausible socioeconomic and emissions scenarios. Environmental Research Letters 10:085010. https://doi.org/10.1088/1748-9326/10/8/085010
